# Crystal structure of 4-acetyl­phenyl 3-methyl­benzoate

**DOI:** 10.1107/S1600536814018923

**Published:** 2014-08-30

**Authors:** Karthik Ananth Mani, Vijayan Viswanathan, S. Narasimhan, Devadasan Velmurugan

**Affiliations:** a Department of Chemistry, Asthagiri Herbal Research Foundation, Perungudi Industrial Estate, Perungudi, Chennai 600 096, India; b Centre of Advanced Study in Crystallography and Biophysics, University of Madras, Guindy Campus, Chennai 600 025, India

**Keywords:** crystal structure, 4-acetyl­phenyl 3-methyl­benzoate, hydrogen bonding, aceto­phenone derivatives

## Abstract

The planes of the aromatic rings of the title compound, C_16_H_14_O_3_, make a dihedral angle of 82.52 (8)°. The acetyl group and the phenyl ring make a dihedral angle of 1.65 (1)°. In the crystal, the molecules are linked by C—H⋯O interactions, generating *C*(7) chains along the *a*-axis direction.

## Related literature   

For the biological activity of aceto­phenone derivatives, see: Chung *et al.* (2003 ▶).
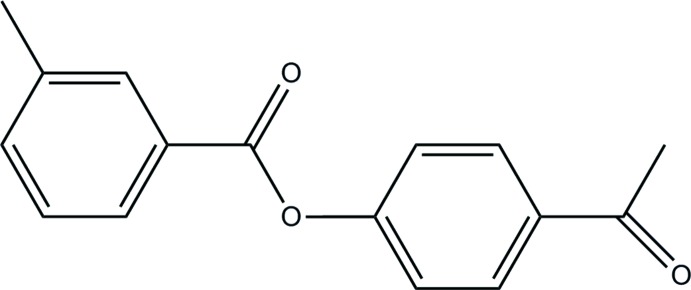


## Experimental   

### Crystal data  

C_16_H_14_O_3_
*M* *_r_* = 254.27
Monoclinic, 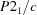

*a* = 8.7167 (3) Å
*b* = 9.8521 (3) Å
*c* = 15.4938 (4) Å
β = 95.149 (2)°
*V* = 1325.20 (7) Å^3^
*Z* = 4
Mo *K*α radiation
μ = 0.09 mm^−1^
*T* = 293 K
0.30 × 0.25 × 0.20 mm

### Data collection  

Bruker SMART APEXII area-detector diffractometer
Absorption correction: multi-scan (*SADABS*; Bruker, 2008 ▶) *T*_min_ = 0.974, *T*_max_ = 0.983
12798 measured reflections
3303 independent reflections
2130 reflections with *I* > 2σ(*I*)
*R*_int_ = 0.033

### Refinement  

*R*[*F* ^2^ > 2σ(*F* ^2^)] = 0.048
*wR*(*F* ^2^) = 0.162
*S* = 1.00
3303 reflections
174 parameters
H-atom parameters constrained
Δρ_max_ = 0.14 e Å^−3^
Δρ_min_ = −0.19 e Å^−3^

### 

Data collection: *APEX2* (Bruker, 2008 ▶); cell refinement: *APEX2*; data reduction: *SAINT* (Bruker, 2008 ▶); program(s) used to solve structure: *SHELXS97* (Sheldrick, 2008 ▶); program(s) used to refine structure: *SHELXL97* (Sheldrick, 2008 ▶); molecular graphics: *ORTEP-3 for Windows* (Farrugia, 2012 ▶); software used to prepare material for publication: *SHELXL97* and *PLATON* (Spek, 2009 ▶).

## Supplementary Material

Crystal structure: contains datablock(s) global, I. DOI: 10.1107/S1600536814018923/bt6992sup1.cif

Structure factors: contains datablock(s) I. DOI: 10.1107/S1600536814018923/bt6992Isup2.hkl

Supporting information file. DOI: 10.1107/S1600536814018923/bt6992Isup3.cml

**Fig. 1.**
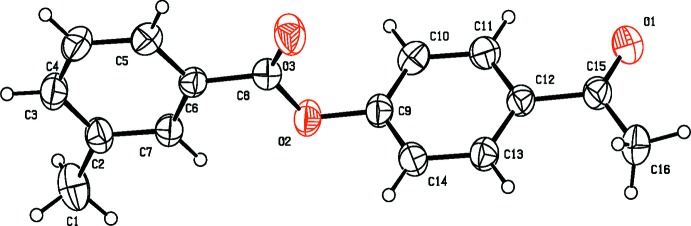
The molecular structure of the title compound, showing the atomic numbering and displacement ellipsoids drawn at 30% probability level.

**Fig. 2.**
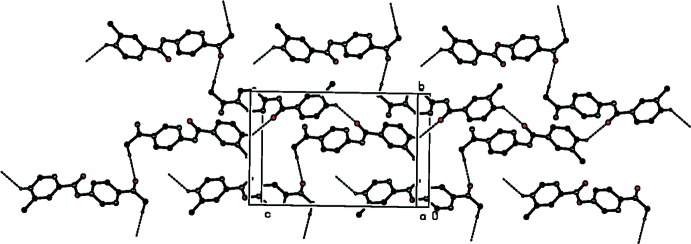
The crystal packing of the title compound viewed down the a axis. Intermolecular hydrogen bonds are shown as dashed lines. H-atoms not involved in H-bonds have been excluded for clarity.

. DOI: 10.1107/S1600536814018923/bt6992fig1.tif
The mol­ecular structure of the title compound, showing the atomic numbering and displacement ellipsoids drawn at 30% probability level.

a . DOI: 10.1107/S1600536814018923/bt6992fig2.tif
The crystal packing of the title compound viewed down the *a* axis. Inter­molecular hydrogen bonds are shown as dashed lines. H-atoms not involved in H-bonds have been excluded for clarity.

CCDC reference: 1020285

Additional supporting information:  crystallographic information; 3D view; checkCIF report

## Figures and Tables

**Table 1:** Hydrogen-bond geometry (Å, °)

*D*—H⋯*A*	*D*—H	H⋯*A*	*D*⋯*A*	*D*—H⋯*A*
C16—H16*B*⋯O1^i^	0.96	2.57	3.509 (3)	167
C3—H3⋯O3^ii^	0.93	2.52	3.265 (2)	137

Symmetry codes: (i) 
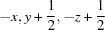
; (ii) 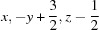.

## supplementary crystallographic information

### S1. Comment

Acetophenone derivatives are popular in organic synthesis for their applications in biology and pharmacological activities. They are known to exhibit antioxidant and antityrosinase activities (Chung *et al.*, 2003).

The ORTEP plot of the molecule is shown in Fig. 1. The carbonyl groups are coplanar with the rings to which they are attached, which is evident from torsion angles [C5-C6-C8-O3 2.1 (2)° and C11-C12-C15-O1 0.9 (2)]. The dihedral angle between the two aromatic rings is 82.52 (8)°.

The molecular structure is stabilized by an intramolecular and the crystal packing by intermolecular C—H···O hydrogen bonds (Table 1 & Fig. 2).

### S2. Experimental

A clean and dry 250ml two neck RB flask was fitted with a condenser and an addition funnel. 0.5 mol of 4- hydroxy acetophenone was taken and 200ml of chloroform was added to it with stirring. The reaction mixture was cooled at 5-10°c. 0.5 mol of meta-tolouyl chloride was added dropwise to the reaction mixture. Stirring was continued for another 15 mins and 0.5 mol of potassium carbonate was slowly added. The reaction was continued for 2 hours and monitored using TLC. The reaction mixture was transferred into a 1 l beaker and washed twice with water (2 x 250 ml). The chloroform layer was separated and washed with 10% NaOH solution (2x250ml). The chloroform layer was separated and dried with anhydrous sodium sulphate. The chloroform layer was then filtered and concentrated under reduced pressure using rotary vacuum. It was cooled and hexane was added to it. The solid which precipitated was filtered and the product was air dried. After purification the compound was recrystallised in CHCl_3_ by slow evaporation method.

### S3. Refinement

The hydrogen atoms were placed in calculated positions with C—H = 0.93Å to 0.96 Å, refined in the riding model with fixed isotropic displacement parameters: *U*_iso_(H) = 1.5*U*_eq_(C) for methyl groups and *U*_iso_(H) = 1.2*U*_eq_(C) for C_aromatic_. The methyl groups were allowed to rotate but not to tip.

### Crystal data

**Table:**

C_16_H_14_O_3_	*F*(000) = 536
*M**_r_* = 254.27	*D*_x_ = 1.274 Mg m^−^^3^
Monoclinic, *P*2_1_/*c*	Mo *K*α radiation, λ = 0.71073 Å
Hall symbol: -P 2ybc	Cell parameters from 3303 reflections
*a* = 8.7167 (3) Å	θ = 2.4–28.3°
*b* = 9.8521 (3) Å	µ = 0.09 mm^−^^1^
*c* = 15.4938 (4) Å	*T* = 293 K
β = 95.149 (2)°	Block, colourless
*V* = 1325.20 (7) Å^3^	0.30 × 0.25 × 0.20 mm
*Z* = 4	

### Data collection

**Table:**

Bruker SMART APEXII area-detector diffractometer	3303 independent reflections
Radiation source: fine-focus sealed tube	2130 reflections with *I* > 2σ(*I*)
Graphite monochromator	*R*_int_ = 0.033
ω and φ scans	θ_max_ = 28.3°, θ_min_ = 2.4°
Absorption correction: multi-scan (*SADABS*; Bruker, 2008)	*h* = −11→9
*T*_min_ = 0.974, *T*_max_ = 0.983	*k* = −10→13
12798 measured reflections	*l* = −20→20

### Refinement

**Table:**

Refinement on *F*^2^	Primary atom site location: structure-invariant direct methods
Least-squares matrix: full	Secondary atom site location: difference Fourier map
*R*[*F*^2^ > 2σ(*F*^2^)] = 0.048	Hydrogen site location: inferred from neighbouring sites
*wR*(*F*^2^) = 0.162	H-atom parameters constrained
*S* = 1.00	*w* = 1/[σ^2^(*F*_o_^2^) + (0.0803*P*)^2^ + 0.1756*P*] where *P* = (*F*_o_^2^ + 2*F*_c_^2^)/3
3303 reflections	(Δ/σ)_max_ = 0.043
174 parameters	Δρ_max_ = 0.14 e Å^−^^3^
0 restraints	Δρ_min_ = −0.19 e Å^−^^3^

### Special details

**Table:**

Geometry. All esds (except the esd in the dihedral angle between two l.s. planes) are estimated using the full covariance matrix. The cell esds are taken into account individually in the estimation of esds in distances, angles and torsion angles; correlations between esds in cell parameters are only used when they are defined by crystal symmetry. An approximate (isotropic) treatment of cell esds is used for estimating esds involving l.s. planes.
Refinement. Refinement of F^2^ against ALL reflections. The weighted R-factor wR and goodness of fit S are based on F^2^, conventional R-factors R are based on F, with F set to zero for negative F^2^. The threshold expression of F^2^ > 2sigma(F^2^) is used only for calculating R-factors(gt) etc. and is not relevant to the choice of reflections for refinement. R-factors based on F^2^ are statistically about twice as large as those based on F, and R- factors based on ALL data will be even larger.

### Fractional atomic coordinates and isotropic or equivalent isotropic displacement parameters (Å^2^)

**Table:**

	*x*	*y*	*z*	*U*_iso_*/*U*_eq_	
C1	0.2306 (3)	1.0754 (3)	−0.43251 (12)	0.0957 (7)	
H1A	0.3009	1.1250	−0.4649	0.144*	
H1B	0.1793	1.1370	−0.3966	0.144*	
H1C	0.1557	1.0305	−0.4718	0.144*	
C2	0.31775 (19)	0.97205 (18)	−0.37669 (9)	0.0637 (4)	
C3	0.4192 (2)	0.88402 (19)	−0.41068 (11)	0.0747 (5)	
H3	0.4342	0.8893	−0.4693	0.090*	
C4	0.4986 (2)	0.7889 (2)	−0.36009 (13)	0.0826 (6)	
H4	0.5666	0.7306	−0.3845	0.099*	
C5	0.4783 (2)	0.77929 (17)	−0.27263 (11)	0.0711 (5)	
H5	0.5326	0.7151	−0.2381	0.085*	
C6	0.37624 (16)	0.86609 (15)	−0.23730 (9)	0.0538 (4)	
C7	0.29747 (17)	0.96191 (16)	−0.28903 (9)	0.0562 (4)	
H7	0.2297	1.0207	−0.2648	0.067*	
C8	0.35490 (18)	0.85016 (16)	−0.14433 (9)	0.0576 (4)	
C9	0.21558 (18)	0.92477 (16)	−0.03042 (9)	0.0581 (4)	
C10	0.10870 (19)	0.83096 (17)	−0.01086 (10)	0.0669 (4)	
H10	0.0630	0.7740	−0.0536	0.080*	
C11	0.06933 (19)	0.82189 (17)	0.07360 (11)	0.0655 (4)	
H11	−0.0027	0.7578	0.0876	0.079*	
C12	0.13591 (17)	0.90698 (15)	0.13718 (9)	0.0553 (4)	
C13	0.24248 (19)	1.00229 (17)	0.11470 (10)	0.0621 (4)	
H13	0.2874	1.0609	0.1567	0.075*	
C14	0.28287 (19)	1.01129 (17)	0.03027 (10)	0.0642 (4)	
H14	0.3545	1.0752	0.0154	0.077*	
C15	0.0930 (2)	0.89341 (17)	0.22797 (11)	0.0662 (4)	
C16	0.1621 (3)	0.9862 (2)	0.29564 (11)	0.0912 (6)	
H16A	0.1261	0.9627	0.3505	0.137*	
H16B	0.1329	1.0779	0.2811	0.137*	
H16C	0.2723	0.9783	0.2993	0.137*	
O1	0.00206 (19)	0.80739 (15)	0.24608 (9)	0.0967 (5)	
O2	0.25094 (14)	0.93822 (12)	−0.11686 (6)	0.0694 (3)	
O3	0.42165 (16)	0.77076 (15)	−0.09685 (7)	0.0882 (4)	

### Atomic displacement parameters (Å^2^)

**Table:**

	*U*^11^	*U*^22^	*U*^33^	*U*^12^	*U*^13^	*U*^23^
C1	0.0939 (14)	0.1320 (18)	0.0614 (10)	0.0018 (13)	0.0082 (9)	0.0211 (11)
C2	0.0628 (9)	0.0782 (11)	0.0507 (8)	−0.0189 (8)	0.0079 (7)	−0.0029 (7)
C3	0.0875 (12)	0.0843 (12)	0.0551 (8)	−0.0229 (10)	0.0220 (8)	−0.0151 (8)
C4	0.0967 (14)	0.0730 (12)	0.0838 (12)	−0.0033 (10)	0.0388 (10)	−0.0202 (10)
C5	0.0796 (11)	0.0615 (10)	0.0747 (10)	0.0042 (8)	0.0199 (9)	−0.0054 (8)
C6	0.0544 (8)	0.0544 (8)	0.0533 (7)	−0.0079 (6)	0.0089 (6)	−0.0067 (6)
C7	0.0549 (8)	0.0630 (9)	0.0513 (7)	−0.0068 (7)	0.0082 (6)	−0.0048 (6)
C8	0.0596 (9)	0.0581 (8)	0.0555 (8)	0.0002 (7)	0.0080 (6)	0.0000 (7)
C9	0.0636 (9)	0.0639 (9)	0.0473 (7)	0.0127 (7)	0.0090 (6)	0.0038 (6)
C10	0.0697 (10)	0.0673 (10)	0.0635 (9)	−0.0004 (8)	0.0047 (8)	−0.0113 (7)
C11	0.0629 (9)	0.0638 (9)	0.0716 (9)	−0.0035 (7)	0.0164 (7)	−0.0027 (8)
C12	0.0558 (8)	0.0564 (8)	0.0550 (8)	0.0108 (6)	0.0115 (6)	0.0026 (6)
C13	0.0682 (9)	0.0672 (9)	0.0511 (7)	−0.0030 (8)	0.0066 (7)	−0.0032 (7)
C14	0.0692 (10)	0.0687 (10)	0.0556 (8)	−0.0057 (8)	0.0107 (7)	0.0051 (7)
C15	0.0742 (10)	0.0623 (9)	0.0652 (9)	0.0159 (8)	0.0240 (8)	0.0063 (7)
C16	0.1263 (17)	0.0948 (14)	0.0551 (9)	0.0026 (13)	0.0238 (10)	−0.0015 (9)
O1	0.1189 (11)	0.0866 (9)	0.0923 (9)	−0.0109 (8)	0.0523 (8)	0.0021 (7)
O2	0.0850 (8)	0.0765 (7)	0.0481 (5)	0.0207 (6)	0.0143 (5)	0.0048 (5)
O3	0.0999 (10)	0.0972 (9)	0.0702 (7)	0.0355 (8)	0.0226 (7)	0.0232 (7)

### Geometric parameters (Å, º)

**Table:**

C1—C2	1.498 (3)	C9—C14	1.362 (2)
C1—H1A	0.9600	C9—C10	1.366 (2)
C1—H1B	0.9600	C9—O2	1.4071 (17)
C1—H1C	0.9600	C10—C11	1.385 (2)
C2—C3	1.377 (2)	C10—H10	0.9300
C2—C7	1.389 (2)	C11—C12	1.381 (2)
C3—C4	1.369 (3)	C11—H11	0.9300
C3—H3	0.9300	C12—C13	1.387 (2)
C4—C5	1.386 (2)	C12—C15	1.494 (2)
C4—H4	0.9300	C13—C14	1.388 (2)
C5—C6	1.382 (2)	C13—H13	0.9300
C5—H5	0.9300	C14—H14	0.9300
C6—C7	1.381 (2)	C15—O1	1.210 (2)
C6—C8	1.477 (2)	C15—C16	1.478 (3)
C7—H7	0.9300	C16—H16A	0.9600
C8—O3	1.1895 (18)	C16—H16B	0.9600
C8—O2	1.3506 (18)	C16—H16C	0.9600
			
C2—C1—H1A	109.5	C14—C9—O2	118.72 (15)
C2—C1—H1B	109.5	C10—C9—O2	119.14 (14)
H1A—C1—H1B	109.5	C9—C10—C11	119.01 (15)
C2—C1—H1C	109.5	C9—C10—H10	120.5
H1A—C1—H1C	109.5	C11—C10—H10	120.5
H1B—C1—H1C	109.5	C12—C11—C10	120.72 (15)
C3—C2—C7	118.12 (16)	C12—C11—H11	119.6
C3—C2—C1	121.15 (15)	C10—C11—H11	119.6
C7—C2—C1	120.73 (16)	C11—C12—C13	118.68 (14)
C4—C3—C2	121.42 (15)	C11—C12—C15	119.52 (14)
C4—C3—H3	119.3	C13—C12—C15	121.80 (14)
C2—C3—H3	119.3	C12—C13—C14	120.81 (14)
C3—C4—C5	120.28 (17)	C12—C13—H13	119.6
C3—C4—H4	119.9	C14—C13—H13	119.6
C5—C4—H4	119.9	C9—C14—C13	118.74 (15)
C6—C5—C4	119.25 (17)	C9—C14—H14	120.6
C6—C5—H5	120.4	C13—C14—H14	120.6
C4—C5—H5	120.4	O1—C15—C16	120.15 (16)
C7—C6—C5	119.82 (14)	O1—C15—C12	120.37 (16)
C7—C6—C8	122.62 (13)	C16—C15—C12	119.48 (15)
C5—C6—C8	117.55 (14)	C15—C16—H16A	109.5
C6—C7—C2	121.10 (14)	C15—C16—H16B	109.5
C6—C7—H7	119.4	H16A—C16—H16B	109.5
C2—C7—H7	119.4	C15—C16—H16C	109.5
O3—C8—O2	122.16 (14)	H16A—C16—H16C	109.5
O3—C8—C6	125.13 (14)	H16B—C16—H16C	109.5
O2—C8—C6	112.70 (13)	C8—O2—C9	116.74 (11)
C14—C9—C10	122.03 (14)		
			
C7—C2—C3—C4	−0.1 (2)	C9—C10—C11—C12	0.5 (2)
C1—C2—C3—C4	−179.69 (18)	C10—C11—C12—C13	0.3 (2)
C2—C3—C4—C5	0.0 (3)	C10—C11—C12—C15	−179.06 (14)
C3—C4—C5—C6	0.4 (3)	C11—C12—C13—C14	−0.7 (2)
C4—C5—C6—C7	−0.7 (2)	C15—C12—C13—C14	178.71 (14)
C4—C5—C6—C8	178.48 (15)	C10—C9—C14—C13	0.8 (2)
C5—C6—C7—C2	0.6 (2)	O2—C9—C14—C13	176.97 (13)
C8—C6—C7—C2	−178.52 (13)	C12—C13—C14—C9	0.1 (2)
C3—C2—C7—C6	−0.2 (2)	C11—C12—C15—O1	0.9 (2)
C1—C2—C7—C6	179.38 (16)	C13—C12—C15—O1	−178.43 (16)
C7—C6—C8—O3	−178.76 (16)	C11—C12—C15—C16	−178.87 (16)
C5—C6—C8—O3	2.1 (2)	C13—C12—C15—C16	1.8 (2)
C7—C6—C8—O2	0.3 (2)	O3—C8—O2—C9	−4.9 (2)
C5—C6—C8—O2	−178.88 (14)	C6—C8—O2—C9	176.01 (13)
C14—C9—C10—C11	−1.1 (2)	C14—C9—O2—C8	100.57 (17)
O2—C9—C10—C11	−177.29 (13)	C10—C9—O2—C8	−83.11 (18)

### Hydrogen-bond geometry (Å, º)

**Table:**

*D*—H···*A*	*D*—H	H···*A*	*D*···*A*	*D*—H···*A*
C7—H7···O2	0.93	2.42	2.7439 (17)	100
C16—H16*B*···O1^i^	0.96	2.57	3.509 (3)	167
C3—H3···O3^ii^	0.93	2.52	3.265 (2)	137

Symmetry codes: (i) −*x*, *y*+1/2, −*z*+1/2; (ii) *x*, −*y*+3/2, *z*−1/2.
