# Supplementary figures and images for: Crystal structure of 4-acetyl­phenyl 3-methyl­benzoate
Source: Acta Crystallogr Sect E Struct Rep Online. 2014 Aug 30;70(Pt 9):o1060. doi: 10.1107/S1600536814018923 (PMC4186166; doi:10.1107/S1600536814018923)

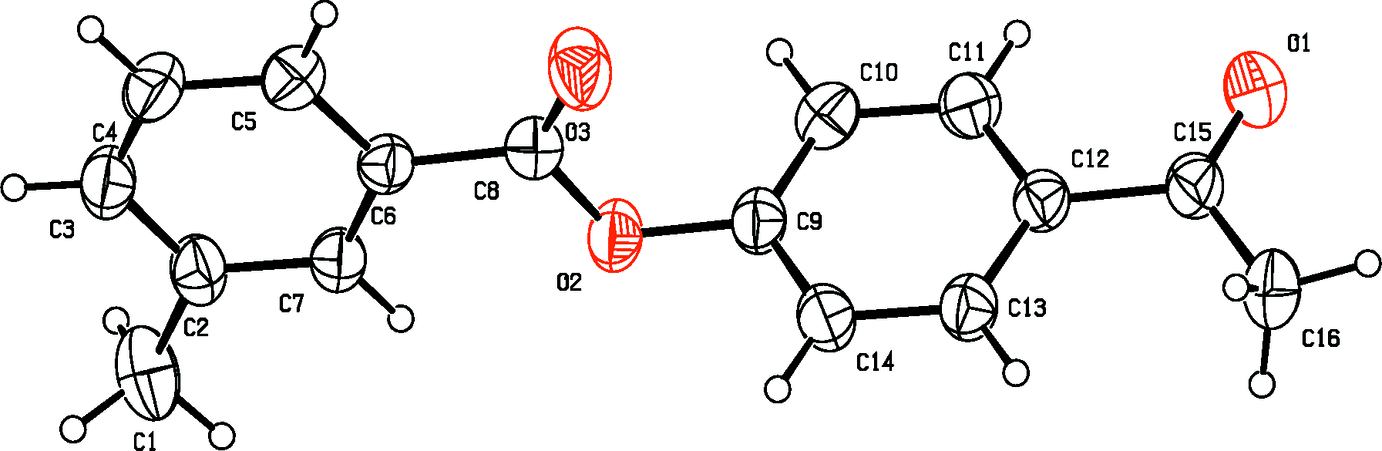

Supplement: Supplementary file 4 [file e-70-o1060-fig1.tif]

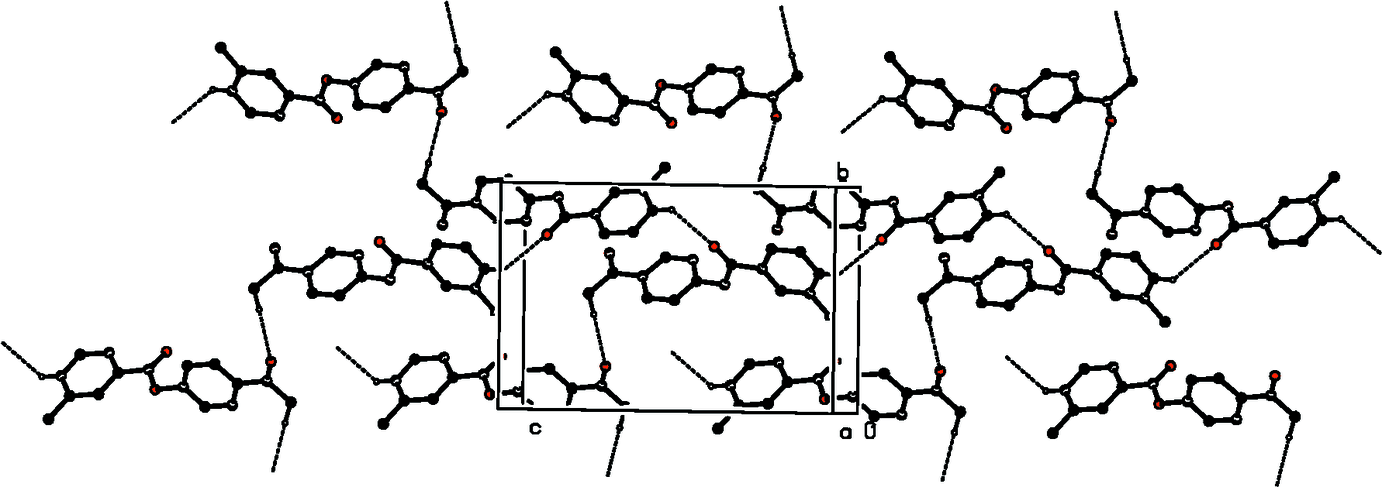

Supplement: Supplementary file 5 [file e-70-o1060-fig2.tif]
